# NLRP3 inflammasome is attenuated in patients with *Mycobacterium avium* complex lung disease and correlated with decreased interleukin-1β response and host susceptibility

**DOI:** 10.1038/s41598-019-47609-3

**Published:** 2019-08-29

**Authors:** Ming-Fang Wu, Chin-Chung Shu, Jann-Yuan Wang, Bo-Shiun Yan, Hsin-Chih Lai, Bor-Luen Chiang, Lawrence Shih-Hsin Wu, Chong-Jen Yu

**Affiliations:** 1 Institute of Statistical Sciences, Academia Sinica, Taipei, Taiwan; 2 Graduate Institute of Toxicology, College of Medicine, National Taiwan University, Taipei, Taiwan; 3 Department of Internal Medicine, National Taiwan University Hospital, Taipei, Taiwan; 4 College of Medicine, National Taiwan University, Taipei, Taiwan; 5 Institute of Biochemistry and Molecular Biology, College of Medicine, National Taiwan University, Taipei, Taiwan; 6 Department of Medical Biotechnology and Laboratory Science, College of Medicine, Chang Gung University, and Research Center for Chinese Herbal Medicine and Research Center for Food and Cosmetic Safety, College of Human Ecology, Chang Gung University of Science and Technology, Tao-Yuan, Taiwan; 7 Department of Pediatrics, National Taiwan University Hospital, Taipei, Taiwan; 8 Graduate Institute of Biomedical Sciences, China Medical University, Taichung, Taiwan

**Keywords:** Tuberculosis, Cell death and immune response

## Abstract

The incidence of nontuberculous mycobacteria lung disease (NTM-LD) is increasing in patients without human immunodeficiency virus. *Mycobacterium avium* complex (MAC) is one of the most common pathogenic species. The presence of MAC has a clinical relevance of around 35~42%, indicating the possibility of host susceptibility. Previous studies have shown that interleukin (IL)-1β and IL-1-receptor knock-out mice are susceptible to mycobacterial infections; however, the role of inflammasome-driven interleukin (IL)-1β has not been studied in MAC-LD. We enrolled patients with MAC-LD and healthy controls. Peripheral blood mononuclear cells (PBMCs), monocytes, and monocyte-derived macrophages were stimulated by MAC bacilli. The responses of interleukin(IL)-1β and the expression of inflammasome and toll-like receptors (TLRs) were measured. Single nucleotide polymorphisms (SNPs) were also examined for *NLRP3* and *TLR2* genes. In the patients with MAC-LD, the IL-1β responses decreased in PBMCs, monocytes, and macrophages assayed by MAC bacilli in comparison to the healthy controls. In addition, the level of caspase-1 after stimulation was lower in the MAC-LD group, although the mRNA level of IL-1β was not significantly lower. In surveying the activation of IL-1β, the MAC-LD group had an attenuated mRNA level of NLRP3 but similar levels of AIM2 and ASC compared with the controls. The SNPs rs3806268 and rs34298354 in *NLRP3* for females and rs3804100 in *TLR2* for males were associated with MAC-LD. In conclusion, our patients with MAC-LD had attenuated IL-1β production, which may have been due to lower activation of the NLRP3-caspase-1 axis. Two SNPs of *NLRP3* and one of *TLR2* were correlated with MAC-LD, possibly indicating host susceptibility.

## Introduction

Lung disease (LD) due to nontuberculous mycobacteria (NTM) has become an important clinical concern^1^ because the incidence and prevalence of NTM-LD has increased over the last 10 years in patients without human immunodeficiency virus^2,3^. *Mycobacterium avium* complex (MAC) is the most common pathogenic species associated with NTM-LD in the USA and South East Asia^2^. The reasons for this increase are not clear; however, it could be related to the increase in patients with acquired compromised immunity and advances in mycobacterial culture techniques^4–7^.

Because MAC exists ubiquitously in the environment, the relevance of sputum growing NTM (the number of patients with true disease over the number of isolated patients) is around 35–42%^8,9^. This indicates host vulnerability; however, the mechanism for it is unknown. During MAC pulmonary infection, macrophages are the first-line defense. Once macrophages sense a variety of inflammatory stimuli, inflammasome receptors form an inflammasome structure and activate the pro-caspase-1 to produce active caspase-1 subunits, which can further process pre-interleukin(IL)-1β into the active cytokine form and induce specific cell death, called pyroptosis^10,11^. The production of IL-1β during a mycobacterial infection is important for successful host immune defense because IL-1β and IL-1-receptor knock-out mice are more susceptible to mycobacterial infections^12–14^.

Inflammasomes are multimeric protein complexes composed of receptors, adaptor proteins and pro-caspase-1, and they are mainly located in innate immune cells^15,16^. Of the inflammasome complexes, nucleotide-binding leucine-rich repeat containing receptor sensor molecule containing pyrin domain 3 (NLRP3) inflammasome is one of the most well reported, and the expression of NLRP3 will be up-regulated once exposed to danger signals or infectious reagents^10,11^ and *Mycobacterium tuberculosis*^17^. The response of NLRP3 inflammasome in macrophages furtherly prevents mycobacterial infection^12,18^. However, the details of the inflammasome response have rarely been investigated in MAC-LD, especially with regards to the defense response and host susceptibility. Therefore, we conducted this study to investigate the role of the NLRP3 inflammasome-driven IL1β axis in MAC-LD.

## Materials and Methods

### Antibodies and reagents

The antibodies for flow cytometry were anti-CD4-APC, anti-CD14-PerCP, and anti-IL-1β-FITC (Biolegend, CA, USA). Isotype-controlled mAbs were also purchased from Biolegend. Recombinant human macrophage colony stimulating factor (M-CSF) and the enzyme-linked immunosorbent assay (ELISA) kits for tumor necrosis factor-alpha (TNF-α), IL-1β, and active caspase-1 were purchased from R&D Systems. All other chemical reagents, unless otherwise specified, were purchased from Sigma-Aldrich (St. Louis, Mo).

### Participant enrollment

This prospective study was conducted at National Taiwan University Hospital in Taiwan from January 2015 to December 2017. The hospital’s Research Ethics Committee approved the study (No. 201308008RINC), and all participants provided written informed consent. All methods were performed in accordance with the relevant ethic guidelines and regulations. Patients aged ≥20 years who had respiratory samples that were culture-positive for MAC were identified. Of these patients, we consecutively enrolled those with MAC-LD according to the ATS diagnostic guidelines^1^. Patients with human immunodeficiency virus (HIV) infection, those co-infected with non-MAC NTM-LD or tuberculosis, and those who had received chemotherapy in the preceding 3 months were excluded. Participants with sputum-negative mycobacterial cultures and normal chest radiographs were enrolled as the controls. All enrolled patients were Han-Taiwanese.

### PBMC and monocytes isolation and differentiation of macrophages

Ten ml whole blood samples from the healthy controls and MAC-LD patients were collected. Peripheral blood mononuclear cells (PBMCs) were immediately collected using Ficoll-Paque PLUS (GE Healthcare). Human CD14^+^ cells were purified from human PBMCs by high-gradient magnetic sorting (Miltenyi Biotec, Bergisch Gladbach, Germany). CD14^+^ monocytes were cultured with 10 ng/ml M-CSF (R&D Systems, MN) for 7 days, and monocyte-derived macrophages (M-Mϕ) were detached for 90 minutes using cell detachment solution (Accutase, Millipore, USA).

### MAC stimulation of cells

*Mycobacterium avium* subspecies *avium* (ATCC 25291) was heated at 80 °C for 30 minutes for inactivation. The heat-killed MAC bacilli were used to stimulate PBMCs or macrophages in the indicated multiplicity of infection at a 1:100 ratio. Lipopolysaccharide (LPS, from *Escherichia coli* 0111: B4; Sigma-Aldrich, USA) was used as the paired positive control. At the indicated time points, the cells or supernatants were collected and further assayed for intracellular cytokine staining, real-time polymerase chain reaction (PCR) or ELISA for cytokine contents, respectively. For the cells used for intracellular staining, we added protein transport inhibitor (BD Bioscience, USA) for the second half of the co-culture.

### RNA isolation and quantitative PCR

Total cellular RNA from M-Mϕ was isolated and first strand cDNA was synthesized. A real-time PCR with iQ SYBR Green Supermix (BIO-RAD, Singapore) was performed using a Bio-Rad MyiQ Single-Color Real-Time PCR Detection System (for details, please see Appendix S3). The mRNA expression level of each target gene was normalized to their respective GAPDH expression. The sequences of sense and anti-sense primers are shown in Table S1 in the Supplement File.

### Flow cytometry analysis

To determine expressions of cell surface markers, cells were re-suspended in FACS buffer (1% FBS in PBS) and incubated with fluorochrome-conjugated antigen-specific mAbs at 4 °C for 30 minutes. To further measure the expression of intracellular cytokines, the cells were fixed and permeabilized using a FIX & PERM™ Cell Permeabilization kit (ThermoFisher Scientific Inc.) and incubated with fluorochrome-conjugated antibodies at 4 °C for 30 minutes. The cell samples were analyzed using FACSVerse (BD Biosciences, USA).

### Western blotting

We prepared cell lysates of PBMC stimulated by dead MAC or infected by viable MAC for 24 hours by lysis buffer (RIPA Lysis Buffer, Millipore, USA) and proteinase inhibitor (Pierce Protease and Phosphatase Inhibitor Mini Tablets, Thermo, USA). The IL-1β and pro-caspase-1 in cell lysates were detected by western blotting analysis (Mini-PROTEAN 3 CELL, Bio-Rad, USA). The blot was incubated with anti-IL-1β antibody (Clone: H-153, SANTA CRUZ BIOTECHNOLOGY, USA) or anti-caspase-1 antibody (Clone: D7F10, Rabbit mAb, Cell Signaling, USA), followed by Biotin-SP (long spacer) AffiniPure Goat Anti-Rabbit IgG (Jackson ImmunoResearch, USA) and then Peroxidase Streptavidin (Jackson ImmunoResearch, USA). For internal control (GAPDH), the membrane was further stripped for 10 minutes by stripping Buffer (GeneMarker, Taiwan) and reprobed with anti-GAPDH antibody (Clone 6C5, Millipore, Germany) and then HRP-conjugated goat anti-mouse IgG antibody (SANTA CRUZ BIOTECHNOLOGY, USA) subsequently.

### DNA extraction and single nucleotide polymorphism analysis

DNA from the PBMCs of consecutively enrolled participants was extracted using a Quick-DNA Miniprep Kit (Zymo Research, CA, USA). Single nucleotide polymorphisms (SNPs) were selected from candidate genes, *AIM2* (absent in melanoma 2), *NLRP3* (nucleotide-binding leucine-rich repeat containing receptor sensor molecule containing pyrin domain 3) and *TLR2* (toll-like receptor 2) according to the study’s findings. By the sample size of the present study, we finally examined SNPs in the exon location and minor allele frequency >1% (information from NCBI dbSNP). Three SNPs in *NLRP3* gene loci, namely, rs34298354, rs3806268 and rs7525979; three SNPs in *TLR2* gene loci, namely, rs3804099, re3804100 and rs5743705; and one each in *caspase-1* (rs1792773) and *AIM2* (rs2276405) were selected to perform genotyping. We used the Agena MassARRAY platform with iPLEX chemistry (Agena, San Diego, CA) to investigate genotyping of the SNPs of interest. The Spectro-CHIPs were analyzed using a MassARRAY Analyzer 4, and the results were analyzed using clustering analysis with TYPER 4.0 software. The allele-specific diagnostic products had a unique molecular weight and were identified using matrix-assisted laser desorption ionization time-of-flight mass spectrometry.

### Statistical analysis

All data are expressed as mean ± standard error or number (%) and were evaluated using the Mann-Whitney *U* test or a paired *t*-test, where appropriate, and the chi-square test, respectively, from the PRISM software package (GraphPad). Odds ratios (ORs) were calculated by logistic regression. We adjusted the ORs of specific SNPs for MAC-LD by age, sex, smoking and underlying immunocompromised diseases. A two-tailed *p* value of <0.05 was considered to be significant.

### Ethics approval and consent to participate

This prospective study was approved by National Taiwan University Hospital’s Research Ethics Committee (No. 201308008RINC), and all participants provided written informed consent.

## Results

### Attenuated IL-1β production in the patients with MAC-LD

**Figure 1:**
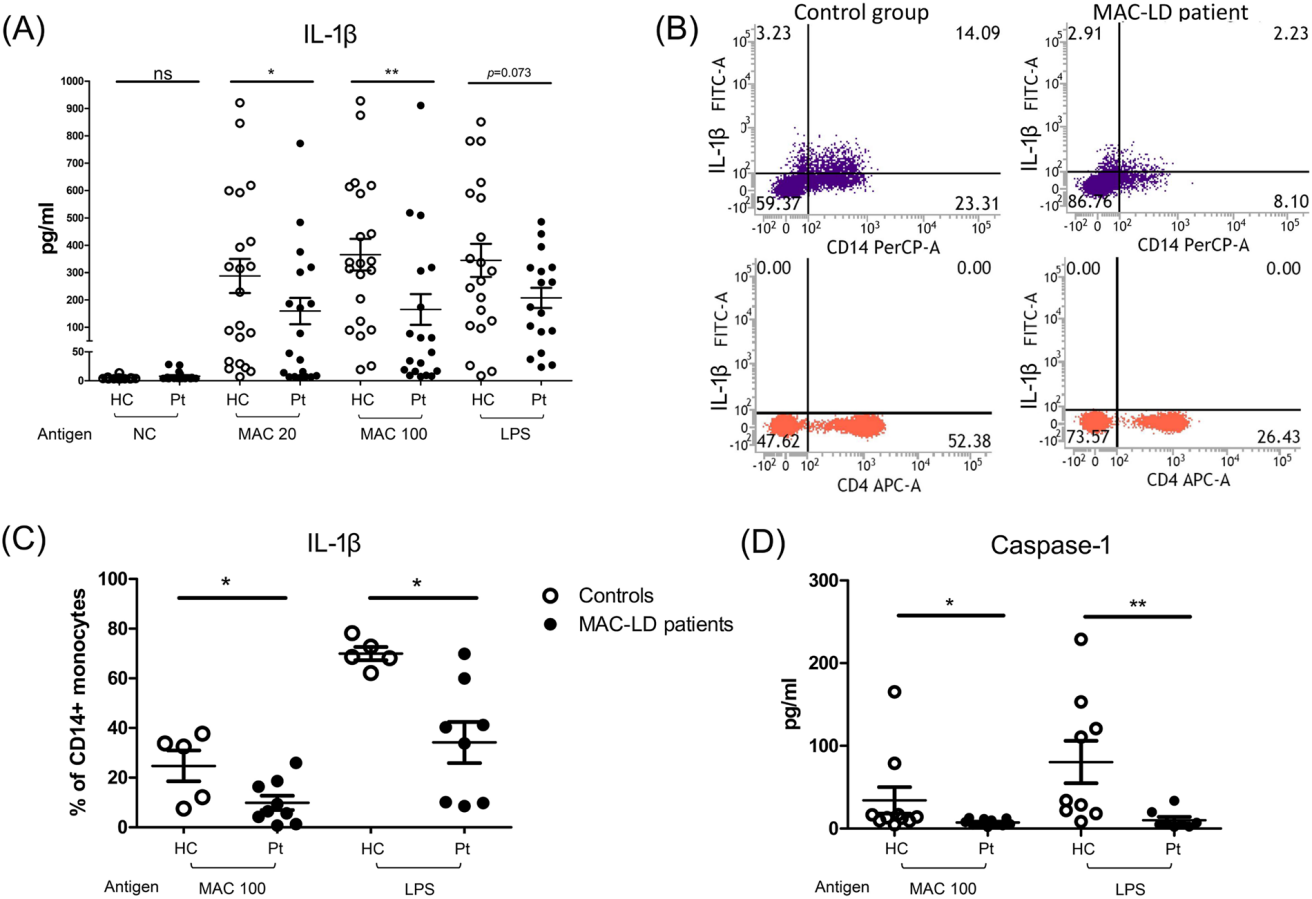
Interleukin 1 beta (IL-1β) and active caspase-1 responses from peripheral blood mononuclear cells (PBMCs) were measured after stimulation with *Mycobacterium avium* complex (MAC) bacilli (multiplicity of infection [MOI] 20 and 100) and lipopolysaccharide (LPS) (5 µg/ml) for 48 hours. (**A**) The levels of secreted IL-1β production from the PBMCs after the indicated stimulation were measured using enzyme-linked immunosorbent assay (ELISA). **(B**) Intracellular IL-1β in the assay was detected using flow cytometry. We discriminated the lymphocytes and monocytes by forward scatter and side scatter. The percentages of IL-1β in CD14 + cells (upper panel) and CD4+ cells (lower panel) were gating in monocyte and lymphocyte populations, respectively. (**C**) The levels of intracellular IL-1β expressed on CD14^+^ monocytes were pooled by dot plots. **(D**) The levels of active caspase-1 from PBMCs assayed after MAC stimulation were measured by ELISA. The data were compared using the Mann Whitney *U* test. The crossed lines are mean values and bars of standard errors. *0.01 ≤ p < 0.05. **0.001 ≤ p < 0.01. ns, not significant; HC, healthy controls; pt, patients.

To study whether MAC-LD infection leads to inflammasome activation in humans, we used MAC to stimulate the PBMCs from age- (42.2 vs 46.3 years, p = 0.251) and sex- (47% vs 33% male, p = 0.520) matched 20 healthy donors and 20 patients with MAC-LD, and found a strong inflammasome activation response with a higher IL-1β release in the healthy controls than in the patients with MAC-LD (290 pg/ml vs 162 pg/ml at MOI = 20; 360 pg/ml vs 178 pg/ml at MOI = 100, respectively). A borderline higher trend of IL-1β release was identified in the healthy controls after the LPS stimulation, a TLR4 agonist (p = 0.073) (Fig. 1A). They did not have underlying disease of malignancy or cirrhosis, and of the MACLD patients, 75% (n = 15) had the nodular-bronchiectasis pattern and 20%, the fibro-cavitary pattern. These results suggested that in contrast to healthy controls, a defective inflammasome platform exists in the PBMCs of patients with MAC-LD.

We then investigated the source of IL-1β in the circulation by detecting the expression of intracellular IL-1β. We found that MAC stimulation caused IL-1β synthesis in CD14^+^ monocytes in both populations, but a lower IL-1β signal in the patients with MAC-LD than in the healthy controls (14.09% vs 2.23%, MOI = 100) (Fig. 1B, upper panel). We did not observe significant expression of IL-1β in CD4^+^ T lymphocytes (Fig. 1B, lower panel). Our observations suggested that monocytes are the target cells in PBMCs for IL-1β synthesis and produced after MAC infection (Fig. 1A,B). The expression of IL-1β in CD14^+^ monocytes was lower in the patients with MAC-LD than in the healthy controls after MAC stimulation (p < 0.05) and LPS treatment (p < 0.05, Fig. 1C). In addition to the synthesis and production of IL-1β, we detected the expression of active caspase-1 to identify the activation of inflammasome and observed a higher expression of active caspase-1 in the control group than in the patients (p < 0.05)(Fig. 1D).

**Figure 2:**
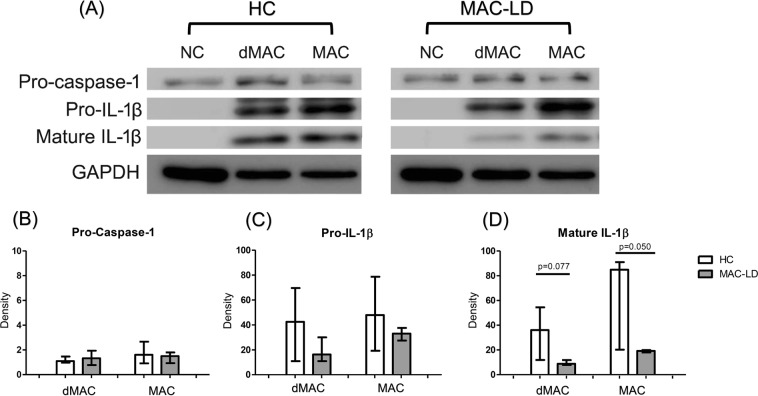
Intracellular protein of interleukin 1 beta (IL-1β) and inactive caspase-1 responses from peripheral blood mononuclear cells (PBMCs) were measured by Western blotting after stimulation with dead *Mycobacterium avium* complex (dMAC) or infection by viable MAC (MAC) bacilli (multiplicity of infection: 100) for 24 hours. (**A**) Case demonstration and (**B**–**D**) bar charts for the protein levels of pro-caspase-1, pro-IL-1β and mature IL-1β by standardizing using individual internal control (GAPDH) and negative control. The data were compared using the Mann Whitney *U* test. The crossed lines are median values and bars of inter-quartile range. HC, healthy controls; LD, lung disease; NC, negative control. Full-length blot is presented in Supplementary Fig. S3.

As shown in Fig. 2, we performed the experiments of western blotting and showed intracellular mature IL-1β response by MAC stimulation, which was borderline attenuated in MAC-LD patients (median: 36.1 vs 8.8, p = 0.077 by dead MAC [n = 3 pairs]; 84.9 vs 19.2, p = 0.050 by viable MAC [n = 3 pairs]). The inactive form of IL-1β (pro- IL-1β) was not significantly different (median: 42.5 vs 16.3, p = 0.400 by dead MAC [n = 3 pairs]; 50.0 vs 33.0, p = 0.686 by viable MAC [n = 3 pairs]). The intracellular inactive caspase-1 (pro-caspase-1) was similar between HC and MAC-LD patients after MAC bacilli stimulation (median: 1.34 vs 13.2, p = 0.686 by dead MAC [n = 3 pairs]; 1.61 vs 1.50, p = 1.000 by viable MAC [n = 3 pairs]) (Fig. 2). Our results suggested that MAC stimulation activated strong inflammasome activation signals in the controls, but that this response was attenuated in the patients with MAC-LD.

### IL-1β protein but not mRNA production was decreased in the monocyte-derived macrophages of the patients with MAC-LD

**Figure 3:**
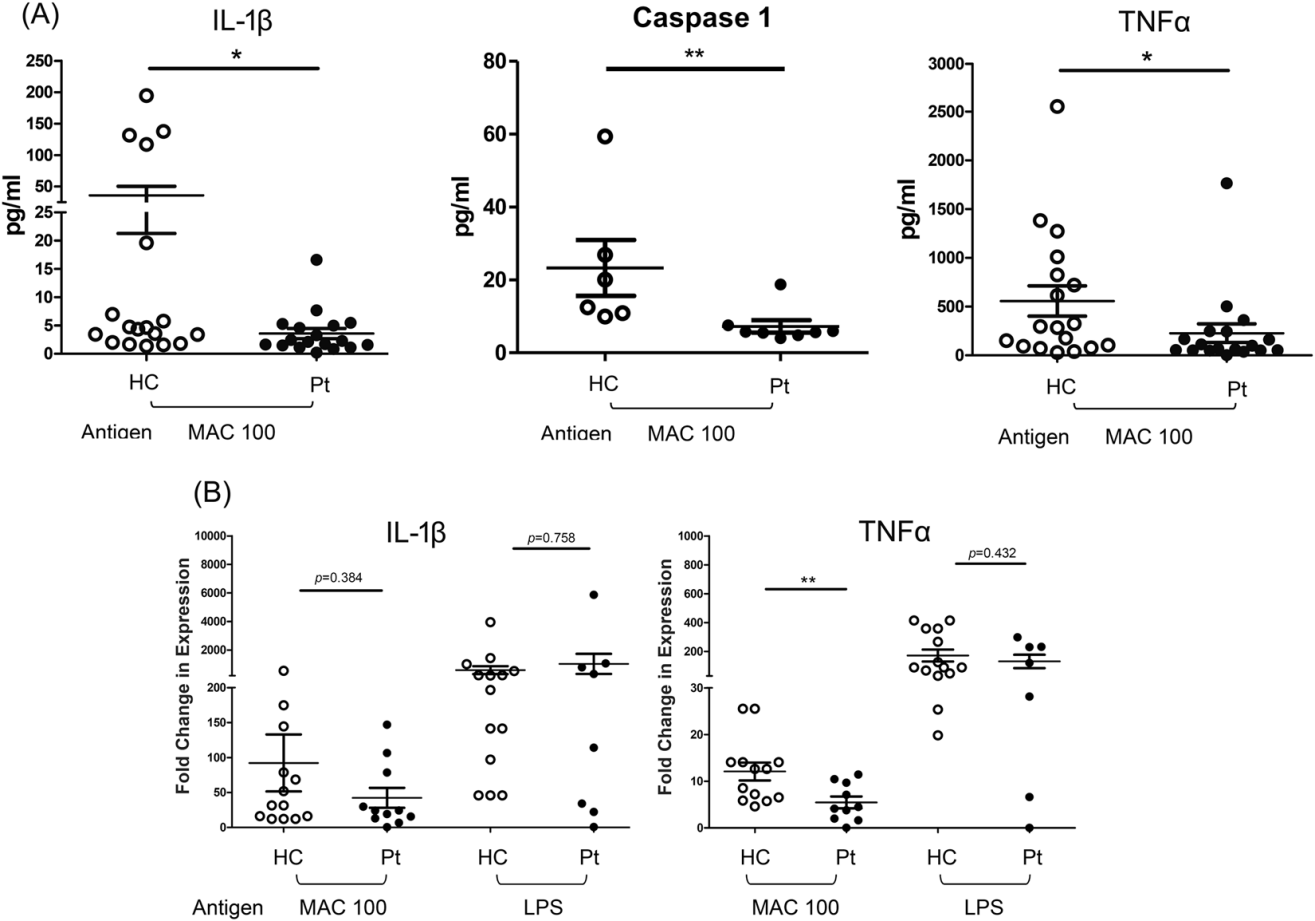
The responses of interleukin 1 beta (IL-1β) and tumor necrosis factor-alpha (TNF-α) were measured in assays of human blood monocyte or its derived macrophages with heat-killed *Mycobacterium avium* complex (MAC) (multiplicity of infection: 100) and lipopolysaccharide (LPS) (5 μg/ml). (**A**) IL-1β and TNF-α in the reaction supernatant by monocyte derived macrophages and caspase-1 from reaction by monocyte, and (**B**) mRNA of cytokines response in cultured cells after stimulation for 24 hours and 6 hours, respectively. The Y axis of mRNA is fold-change compared to the negative controls. The data were compared using the Mann Whitney *U* test. The crossed lines are mean values and bars of standard errors. *0.01 ≤ p < 0.05. **0.001 ≤ p < 0.01. HC, healthy controls; pt, patients.

When humans are infected with pathogens, macrophages act against locally invading pathogens in the lungs. Accordingly, we used the *in-vitro* monocyte/macrophage system and stimulated monocyte or M-Mϕ with MAC bacilli. Higher production of IL-1β (40 pg/ml vs 5 pg/ml, p < 0.05, from M-Mϕ) (Fig. 3A, left panel), active caspase-1 (23.3 pg/ml vs 7.3 pg/ml, p = 0.005, from monocyte) (Fig. 3A, middle panel) and TNF-α (500 pg/ml vs 200 pg/ml, p < 0.05, from M-Mϕ) (Fig. 3A, right panel) were induced from the healthy controls than from that derived from the patients with MAC-LD.

In addition to caspase-1 activation, IL-1β transcription is another key factor for successful IL-1β production^10^. We studied whether the priming signal for IL-1β RNA synthesis is defective in patients with MAC-LD. The results showed that there was no significant difference in IL-1β transcripts for both groups (controls vs MAC-LD group) after MAC stimulation (p = 0.364), indicating that the priming signals may be similar in both groups (Fig. 3B). In contrast, we observed a positive correlation between RNA transcripts and cytokines in TNF-α for both groups after MAC stimulation (Fig. 3A,B, right panel).

### Expressions of NLRP3 and TLR2 were lower in the patients with MAC-LD than in the healthy controls

**Figure 4:**
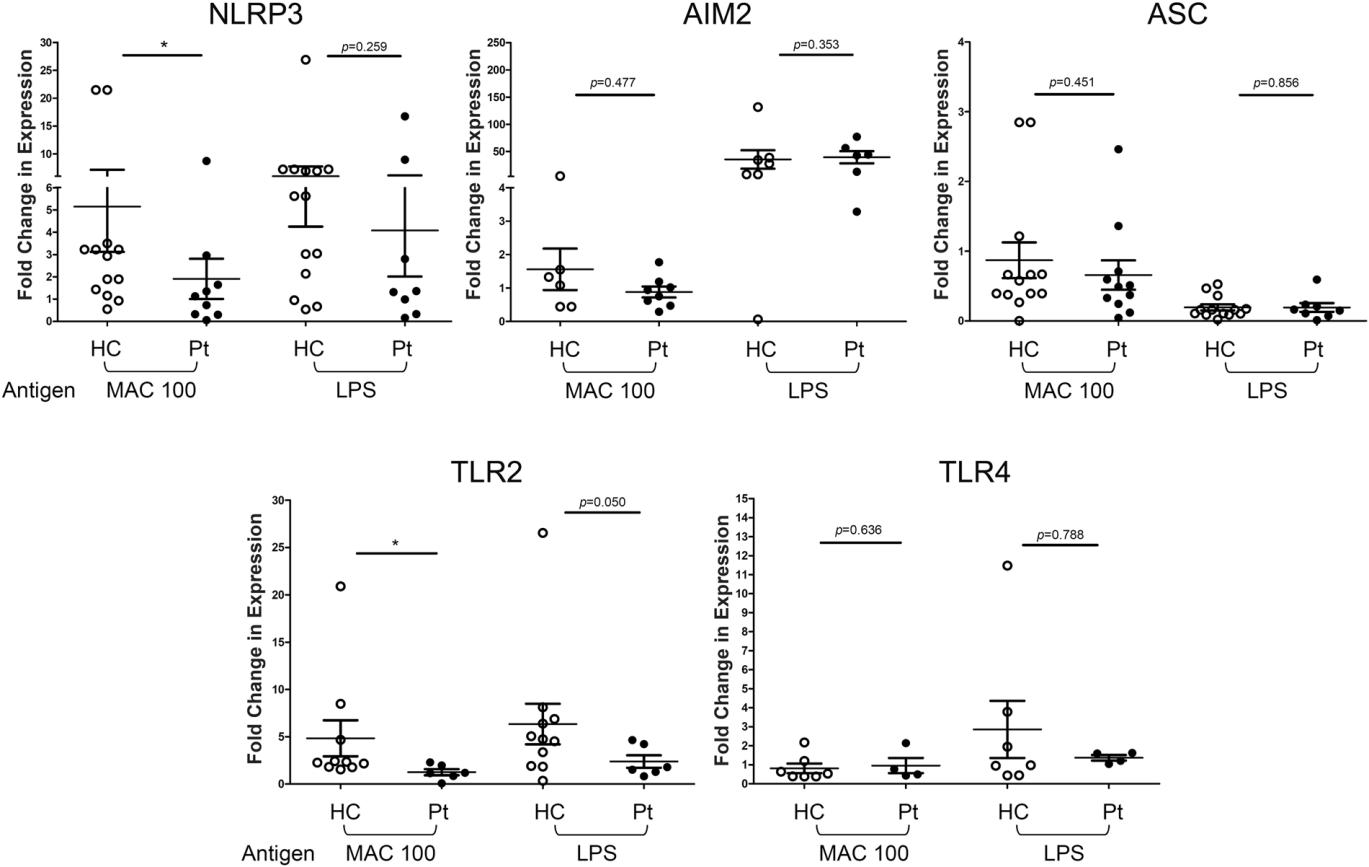
The mRNA levels of inflammasome receptors including NLRP3 and AIM2, adaptor protein-like ASC, and toll-like receptor 2 (TLR2) and TLR4 were measured in assays of human blood monocyte-derived macrophages with heat-killed *Mycobacterium avium* complex (MAC) (multiplicity of infection: 100) and lipopolysaccharide (LPS) (5 μg/ml) for 6 hours. The data were compared using the Mann Whitney *U* test. The crossed lines are mean values and bars of standard errors. *0.01 ≤ p < 0.05. **0.001 ≤ p < 0.01. HC, healthy controls; pt, patients.

We determined the increase in expression of inflammasome receptors and adaptor proteins human M-Mφ, which may have controlled the activation of inflammasomes after MAC stimulation. The increased in the expression of NLRP3 on M-Mφ was greater in the control group than in the MAC-LD group after MAC stimulation, but there were no significant differences in other inflammasome receptors, including AIM2 and apoptosis-associated speck-like protein containing a CARD (ASC) between the two groups (Fig. 4). On the other hand, LPS stimulation led to insignificant differences in NLRP3 and AIM2 receptors between both groups. For TLR, the message RNA expression of TLR2 after MAC bacilli stimulation was lower in the MAC-LD group, whereas the level of TLR4 was similar (Fig. 4). The correlation was significant between mRNA expression of NLRP3 and the protein production of IL1-β from M-Mφ stimulated by MAC bacilli (Pearson correlation: 0.368, p = 0.029).

### SNPs of *NLRP3* and *TLR2* genes were associated with MAC-LD

As defective caspase-1 activation (Fig. 1D) and lower NLRP3 and TLR2 transcription (Fig. 4A) were found in the patients with MAC-LD, we tried to identify whether there was a differential SNP pattern for the genes that were correlated with reduced IL-1β production. We enrolled 106 patients with MAC-LD who were older (65.7 ± 12.6 vs 57.1 ± 15.3 years, p < 0.001) than the 119 controls, but they had a similar male proportion (40% vs 48%, p = 0.212). There were no significant between-group differences regarding smoking status and underlying immunocompromised diseases including diabetes mellitus, cancer, and autoimmune disease (for details, please see Table S3 in the Supplement File). We analyzed three SNPs in *NLRP3* gene loci, namely, rs34298354, rs3806268 and rs7525979; three SNPs in *TLR2* gene loci, namely, rs3804099, rs3804100 and rs5743705; and one each in *caspase-1* (rs1792773) and *AIM2* (rs2276405) (Table 1). Among them, the pattern of GA in rs3806268, compared with GG and AA, was associated with MAC-LD (p = 0.022, Fisher’s exact test). In logistic regression analysis, the crude odds ratio (OR) for MAC-LD was 1.782 (95% CI 1.049–3.027, p = 0.033), and the OR adjusted by age, sex, smoking, and underlying disease was 1.788 (95% CI: 1.022–3.130, p = 0.042).

**Table 1:** The analysis of single nucleotide polymorphisms (SNPs).

SNP	Location	p value for HWE	Genotype	All			Male			Female		
				No.	% in with MAC-LD	p value	No.	% with MAC-LD	p value	No.	% with MAC-LD	p value
rs1792773	CARD17	0.4726	TT	41	21 (51)	0.831	20	10 (50)	0.546	21	11 (52)	0.741
	5′ UTR		TC	120	56 (47)		49	18 (37)		71	38 (54)	
			CC	64	29 (45)		31	14 (45)		33	15 (46)	
rs2276405	AIM2	0.6921	TC	26	13 (50)	0.613	12	7 (58)	0.222	14	6 (43)	0.463
	Exonic stop gain		CC	198	93 (47)		88	35 (40)		110	58 (53)	
rs34298354	NLRP3	0.6985	CT	25	13 (56)	0.345	16	6 (38)	0.691	9	8 (89)	0.019
	Exonic synonymous		CC	200	92 (46)		84	36 (43)		116	56 (48)	
rs3806268	NLRP3	0.7203	AA	77	32 (42)	0.096	30	11 (37)	0.515	47	21 (45)	0.153
	Exonic		GA	104	57 (55)		48	23 (48)		56	34 (61)	
	synonymous		GG	44	17 (39)		22	8 (36)		22	9 (41)	
rs7525979	NLRP3	0.9995	CC	166	79 (48)	0.511	71	30 (42)	0.340	95	49 (52)	0.589
	Exonic		CT	54	24 (44)		25	9 (36)		29	15 (52)	
	synonymous		TT	4	3 (75)		4	3 (75)		0		
rs3804099	TLR2	0.6884	TT	106	54 (51)	0.582	47	24 (51)	0.088	59	30 (51)	0.094
	Exonic		TC	100	44 (44)		42	17 (41)		58	27 (47)	
	synonymous		CC	18	8 (44)		10	1 (10)		8	7 (88)	
rs3804100	TLR2	0.1096	CC	10	2 (20)	0.224	7	0	0.022	3	2 (67)	0.657
	Exonic		CT	97	45 (46)		40	14 (35)		57	31 (54)	
	synonymous		TT	117	59 (50)		52	28 (54)		65	31 (48)	
rs5743705	TLR2	0.6776	CT	25	13 (52)	0.603	11	5 (46)	0.806	14	8 (57)	0.637
	Exonic synonymous		CC	200	93 (47)		89	37 (42)		111	56 (51)	

Genotyping missing: 1 in rs2276405, 1 in rs3804100, 1 in rs3804099, 1 in rs7525979.

Abbreviation: HWE, Hardy Weinberg Equilibrium.

Furthermore, we investigated associations between interactions of genetic variants and sex. If we stratified the population by sex, the GA of rs3806268 (p = 0.041, Fisher’s exact test) and CT in rs34298354 (p = 0.019) were correlated with the disease in the female population. The crude OR of MAC-LD was 2.009 (95% CI: 0.981–4.115, p = 0.072) and the adjusted OR was 1.945 (95% CI: 0.913–4.147, p = 0.085) for GA of rs3806268. For CT in rs34298354, the crude and adjusted ORs were 8.571 (95% CI: 1.039–70.738, p = 0.033) and 8.517 (95% CI: 1.010–71.838, p = 0.049), respectively. With regards to the *TLR2* polymorphism, TT in rs3804100 favored MAC-LD with a crude OR of 2.750 (95% CI: 1.200–6.304, p = 0.025) and an adjusted OR of 3.050 (95% CI: 1.218–7.638, p = 0.017) in the male population. Taken together, these results suggest that *NLRP3* and *TLR2* polymorphisms may influence the genetic susceptibility to MAC-LD according to sex. The genetic variants of *AIM2* did not have any associations with MAC-LD.

**Figure 5:**
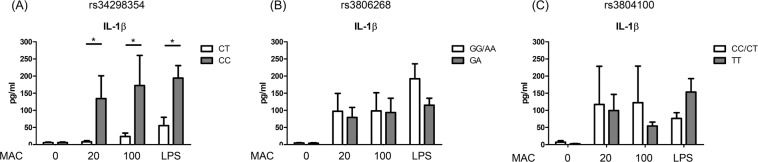
Under different patterns of single nucleotide polymorphisms, we measured and compared interleukin 1-beta (IL-1β) responses from peripheral blood mononuclear cells (PBMCs) after stimulation with dead *Mycobacterium avium* complex (MAC) bacilli (multiplicity of infection [MOI] 20 and 100) and lipopolysaccharide (LPS) (5 µg/ml) for 48 hours. The data were compared using the Mann Whitney *U* test. The crossed lines are mean values and bars of standard errors. *0.01 ≤ p < 0.05.

For eighteen subjects, IL-1β ELISA and genotyping for genetic variants of *NLRP3*/*TLR2* were performed. Among them, the SNP rs34298354 in the *NLRP3* exon region showed significant association with IL-1β production in ten female subjects (Fig. 5A). For the SNP rs3806268 in *NLRP3* for all eighteen subjects (Fig. 5B) and rs3804100 in *TLR2* for eight male subjects (Fig. 5C), there were no significant associations with IL-1β response in PBMC stimulation.

## Discussion

In the present study, the *in-vitro* response of IL-1β after MAC stimulation was decreased in the patients with MAC-LD compared to the healthy controls. The level of caspase-1 after the stimulation was lower in the patients with MAC-LD, but the mRNA expression of IL-1β was similar between the two groups. For the activation of inflammation, NLRP3 mRNA was attenuated in the patients, which correlated with lower IL-1β expression. For SNP analysis, the rs3806268 and rs34298354 SNPs in *NLRP3* and rs3804100 in *TLR2* were associated with MAC-LD. Among them, rs34298354 was directly associated with IL-1β response.

NLRP3 can respond to a range of intracellular damage or pathogen-derived ligands and then activate inflammasome assembly, thereby inducing the production of pro-inflammatory cytokines^19^. Mycobacteria infection initiates the NLRP3-dependent IL-1β axis^20^, which restricts the growth of mycobacteria^18^. To date, few studies have investigated the association between inflammasomes and MAC bacilli, which is one of the most common species involved in NTM-LD. We found that MAC bacilli could activate NLRP3 inflammasomes and downstream IL-1β production. However, the activations of NLRP3, caspase-1 and IL-1β were reduced in the patients with MAC-LD. This may indicate a susceptibility to MAC, although the detailed mechanism for the reduced response is unknown at present. A previous study suggested that *Mycobacterium tuberculosis* can suppress NLRP3-related innate immunity and favor evasion from macrophages^21^. The differences in the NLRP3-IL-1β axis between the patients with MAC-LD and controls suggested that the patients probably had immune vulnerability to MAC bacilli, leading to disease progression after exposure to environmental NTM^22^. However, the distributions of IL-1β were overlapped in the controls and patients (Figs 1A, 3A), and the higher trend in the controls was influenced by only a proportion of the subjects with higher expression. This finding indicates that the inflammasome influencing IL-1β exists in a proportion of MAC-LD patients but not in every patient.

In addition to a low response of NLRP3-dependent IL-1β, we also observed a low caspase-1 response in the MAC-LD group, suggesting that attenuated pyroptosis may exist in MAC-LD. Pyroptosis, a conserved program of inflammatory cell death^23^, is accompanied by caspase-1-dependent processing and activation of the inflammatory cytokine IL-1β^24^. With low pyroptosis, intracellular MAC bacilli may not be cleaned and then survive in macrophages^25^.

In the host genetic study, SNP analysis of the *NLRP3* and* TLR2* genes revealed that rs3806268, an SNP of *NLRP3*, had a GA variant associated with MAC-LD compared with a homo-dimer in the SNP locus. A CT variant of rs34298354 in *NLRP3* was significantly associated with MAC-LD in the female subgroup. *NLRP3* gene polymorphisms have been associated with inflammation-related diseases such as inflammatory bowel disease^26^, juvenile idiopathic arthritis and type 2 diabetes^27^; however, few studies have investigated immune dysfunction. The association between SNPs of the *NLRP3* gene and MAC-LD in the females may indicate more direct evidence of host susceptibility to MAC bacilli. However, further studies are needed to investigate the dysfunction of the corresponding gene locus and the mechanism for the hypo-response of the NLRP3 axis to MAC infection.

The SNP rs34298354 (C2054T) is exonic polymorphism of the *NLRP3* gene and is a silent polymorphism. The synonymous SNP is translated to the 436^th^ amino acid in the NLRP3 protein. Codon usage for the synonymous SNP rs34298354, with TCC changed to TCT (both encode serine), changes from 22% (frequency per thousand: 17.7) to 19% (frequency per thousand: 17.2; codon usage database: https://www.kazusa.or.jp/codon/cgi-bin/showcodon.cgi?species=9606). Previous reports of the Multidrug Resistance 1 (MDR1) gene indicated that silent polymorphism can change the substrate specificity^28,29^. The SNP rs34298354 in the *NLRP3* gene, associated with IL-1β production, may have a function like the MDR1 gene’s silent polymorphism, but it needs to be further elucidated.

The trend of IL-1β mRNA was not significantly different between the control and patient groups, but TLR2 mRNA decreased significantly in the patient group compared with the controls. The possible explanations might be that IL-1β production could be regulated by many pathways^13,30^ other than TLR2 signaling. The TLR2 mRNA in MAC-LD patients was significantly decreased, but possibly not enough to make IL-1β mRNA decrease significantly in a small case number. Other pathways were not studied in the present study and need to be investigated in the future.

The response of the mRNA of the TLR2 and TNFα axis to MAC was also impaired in the patients, which is consistent with previous reports^31,32^. Some gene polymorphisms of *TLR2* have been reported^33,34^; however, the current study is the first to report a correlation between a *TLR2* polymorphism and male MAC-LD. This finding suggests that multi-factorial predisposing factors cause MAC-LD, and that a different pathogenesis may exist in different genders.

There are several limitations to the present study. First, the case number was small and some significance may have been under-estimated. Second, the cases were enrolled in Taiwan, and the results may not be generalized to other ethnicities or areas.

## Conclusion

The responses of IL-1β in macrophages/monocytes were attenuated in the patients with MAC-LD. This may have been due to an impaired expression of NLRP3 inflammasome, which is responsible for the activation process of IL-1β. The gene polymorphisms of *NLRP3* and *TLR2* were correlated with MAC-LD, suggesting a possible host vulnerability. Further studies are needed to investigate the details of the impaired mechanism between gene polymorphisms and protein levels.

## Supplementary information

Supplemental file

## Data Availability

The data of the present study will not be shared because no permission was granted by the Research Ethics Committee.
