# Supplementary material for: NLRP3 inflammasome is attenuated in patients with Mycobacterium avium complex lung disease and correlated with decreased interleukin-1β response and host susceptibility
Source: Sci Rep. 2019 Aug 29;9:12534. doi: 10.1038/s41598-019-47609-3 (PMC6715708; doi:10.1038/s41598-019-47609-3)
Supplement: Supplementary file 1 — Supplemental file [file 41598_2019_47609_MOESM1_ESM.doc]

**SUPPLEMENTARY INFORMATION**

**Title: NLRP3 inflammasome is attenuated in patients with *Mycobacterium avium* complex lung disease and correlated with decreased interleukin-1β response and host susceptibility**

Ming-Fang Wu^1,2^*, Chin-Chung Shu^3,4^*, Jann-Yuan Wang^3,4^, Bo-Shiun Yan^5^, Lai Hsin-Chih^6^, Bor-Luen Chiang^4,7^, Lawrence Shih-Hsin Wu^8#^, Chong-Jen Yu^3,4#^

**Institutions:**

^1^Institute of Biomedical Sciences, Academia Sinica, Taipei, Taiwan

^2^Graduate Institute of Toxicology, College of Medicine, National Taiwan University, Taipei, Taiwan

^3^Department of Internal Medicine, National Taiwan University Hospital, Taipei, Taiwan

^4^College of Medicine, National Taiwan University, Taipei, Taiwan

^5^Graduate Institute of [Biochemistry and Molecular Biology](http://www.mc.ntu.edu.tw/department/ibmb/IBMB/index.html), College of Medicine, National Taiwan University, Taipei, Taiwan

^6^Department of Medical Biotechnology and Laboratory Science, College of Medicine, Chang Gung University, and Research Center for Chinese Herbal Medicine and Research Center for Food and Cosmetic Safety, College of Human Ecology, Chang Gung University of Science and Technology, Tao-Yuan, Taiwan

^7^Department of Pediatrics, National Taiwan University Hospital, Taipei, Taiwan

^8^Graduate Institute of Biomedical Sciences, China Medical University

^#^, *These two authors contributed equally to this work.

**Corresponding authors**:

Chong-Jen Yu, MD., PhD.

Department of Internal Medicine, National Taiwan University Hospital,

No. 7, Chung Shan South Road, Taipei, Taiwan; E-mail: [jefferycjyu@ntu.edu.tw](mailto:jefferycjyu@ntu.edu.tw)

And

Lawrence Shih-Hsin Wu

Graduate Institute of Biomedical Sciences, China Medical University

No. 91, Hsueh-Shih Road, Taichung, Taiwan; Email: lshwu@hotmail.com

**Running Title:** NLRP3 in MAC-LD susceptibility

**Appendix S1- PBMC isolation**

Ten ml whole blood samples from the healthy controls and MAC-LD patients were collected. Peripheral blood mononuclear cells (PBMCs) were immediately collected using Ficoll-Paque PLUS (GE Healthcare), washed twice with phosphate-buffered saline (PBS) and frozen using Cell Banker (ZENOAQ, Japan) at -80°C following the manufacturers’ instructions. The PBMCs were defrosted within days for the scheduled experiments. Dead cells were ruled out using the 0.4% trypan blue staining method.

**Appendix S2- RNA isolation and quantitative PCR**

Total cellular RNA from M-Mφ was isolated using a Direct-zol™ RNA MiniPrep kit (Zymo Research, CA, USA) based on the manufacturer’s instructions. First, strand cDNA was synthesized using the an iScript^TM^ cDNA Synthesis kit (Life Science Research, USA) according to the manufacturer’s instructions. A real-time PCR with iQ SYBR Green Supermix (BIO-RAD, Singapore) was performed using a Bio-Rad MyiQ Single-Color Real-Time PCR Detection System and analyzed using a Bio-Rad iQ5 Optical System 2.0 (Bio-Rad, CA). The PCR mixtures were denatured at 95°C for 10 min, followed by 45 cycles of 15 seconds at 95°C, 20 seconds at 58°C, and 30 seconds at 72°C for amplification. The mRNA expression level of each target gene was normalized to their respective GAPDH expression.

**Appendix S3- Single nucleotide polymorphism analysis**

According to the manufacturer’s instructions, the specific PCR primer and extension primer sequences (Table S2 in the supplement file) were designed using the Assay Designer software package (v.4.0). We used 1 µl of genomic DNA sample (10 ng/µl) for the multiplex PCR in 5-µl volumes containing 1 unit of Taq polymerase, 500 nmol of each PCR primer mix and 2.5 mM of each dNTP (Agena, PCR Accessory and Enzyme kit). Thermocycling was performed at 94°C for 4 minutes followed by 45 cycles at 94°C for 20 seconds, 56°C for 30 seconds, 72°C for 1 minute, and 72°C for 3 minutes. Unincorporated dNTPs were deactivated using 0.3 U of shrimp alkaline phosphatase. Single base extension reactions were performed using iPLEX enzyme, terminator mix, and extension primer mix followed by 94°C for 30 seconds followed by 40 cycles at 94°C for 5 seconds, 5 inner cycles at 56°C for 5 seconds, 80°C for 5 seconds, and 72°C for 3 minutes (Agena, iPLEX kit). After the addition of a cation exchange resin to remove residual salt from the reactions, 7 nl of the purified primer extension reaction was loaded onto the matrix pad of a Spectro-CHIP (Agena). The Spectro-CHIPs were analyzed using a MassARRAY Analyzer 4, and the calling was analyzed using clustering analysis with TYPER 4.0 software. We amplified the DNA containing the SNPs of interest. The allele-specific diagnostic products had a unique molecular weight and were identified using matrix-assisted laser desorption ionization time-of-flight mass spectrometry.

**Figure S1.** The correlation, shown by dot plot, is analyzed between the mRNA level of *NLRP3* and the protein production of IL1-β from M-Mφ stimulated by *Mycobacterium avium* complex (MAC) bacilli (multiplicity of infection: 100). The statistics of correlation uses Pearson correlation.


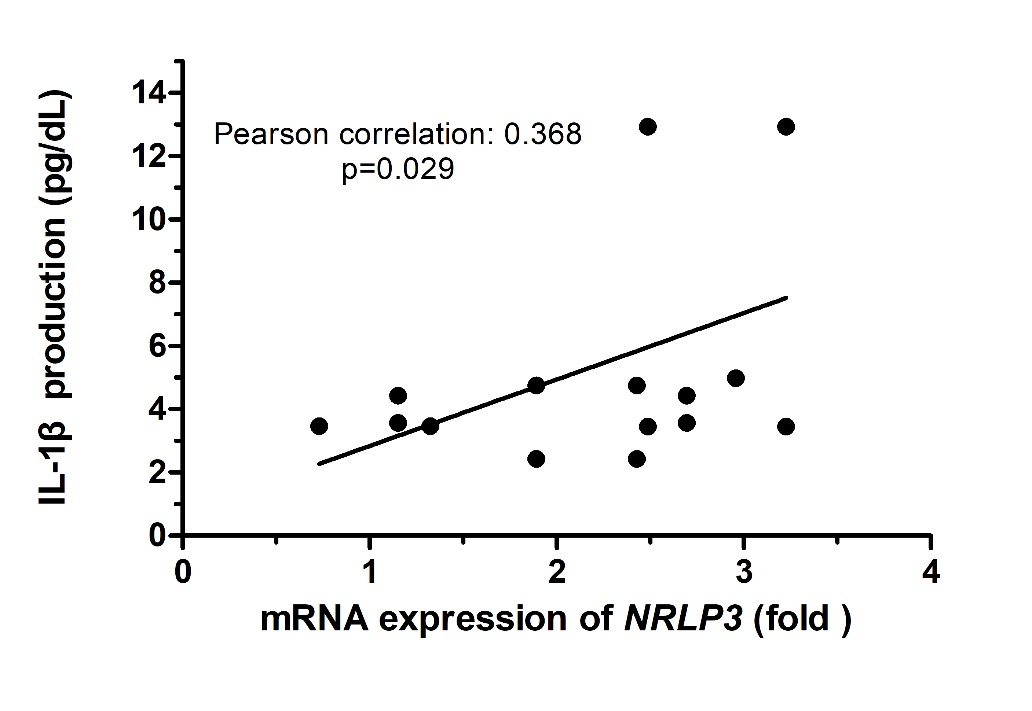


**Figure S2.** The interleukin-1beta (IL-1β) response to 24 hours of *Mycobacterium avium* complex (MAC) co-culturing with THP-1 cells. The experiments are repeated for three times and shown by dot plot. Dead MAC bacilli (dMAC) are applied with multiplicity of infection (MOI) of 10, 30, and 100 whereas live MAC with MOI of 100. We use paired t test for the statistics of comparison. *p value< 0.05, and 0.001<**<0.01. The response by lipopolysaccharide (5 μg/ml) is higher than every other group (not shown in the figure due to limited space).

**Figure S3.** The western blots in Figure 2 shows bands of pro-caspase-1, pro-interleukin-1β (IL1-β), and mature IL-1β were come from the same original blot but cropped into three pieces for easy performing immune blotting. dMAC, dead *Mycobacterium avium* complex; MAC, viable *Mycobacterium avium* complex; HC, healthy controls; LD, lung disease; NC, negative control.


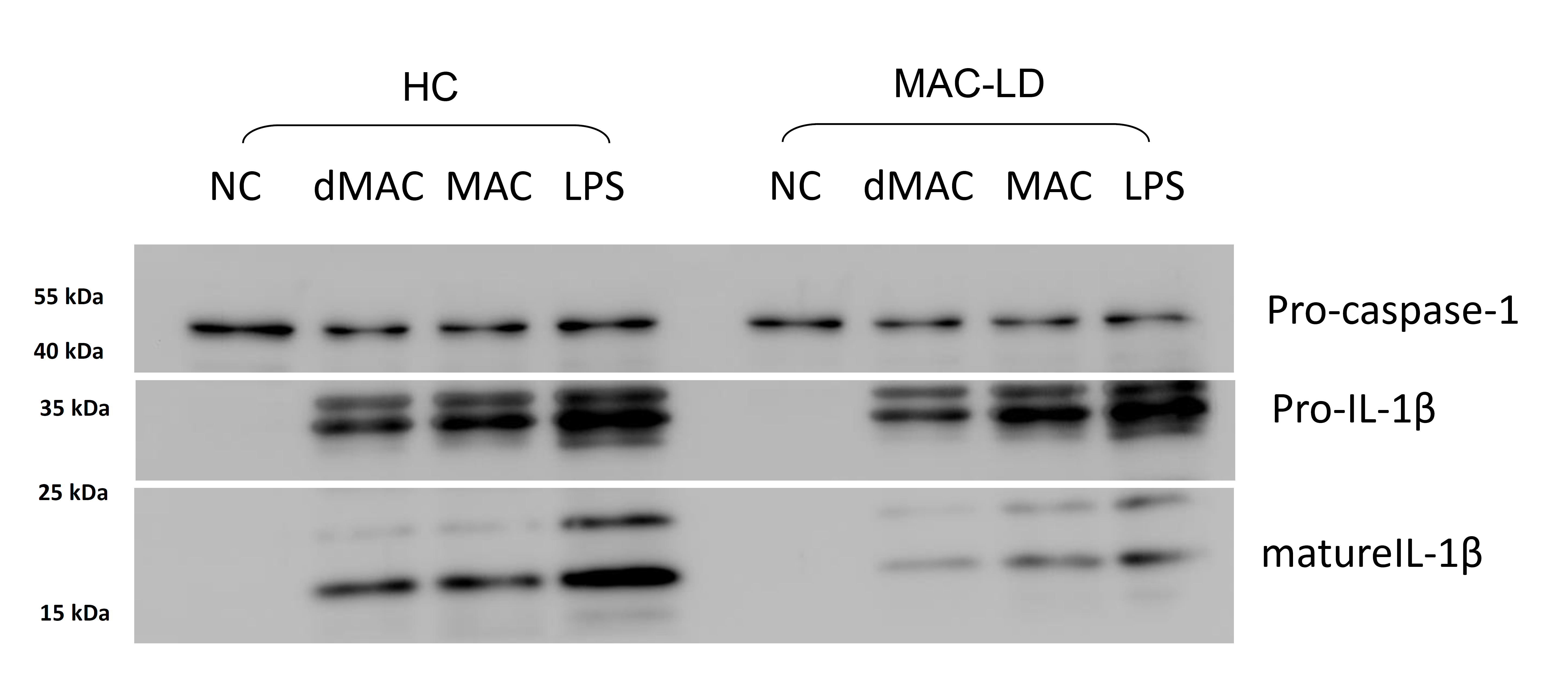


**Table S1** Primers for real time polymerase chain reaction.

| **Primers** | **Forward (5’-3’)** | **Reverse (5’-3’)** |
| --- | --- | --- |
| **TNF-α** | CTTGTTCCTCAGCCTCTTCTCCTT | AACATGGGCTACAGGCTTGTCACT |
| **IL-1β** | GGATATGGAGCAACAAGTGG | GAAGTCAGTTATATCCTGGC |
| **NLRP3** | ACTCTGTGAGGGACTCTTGC | GGTCGCCCAGGTCATTGTT |
| **AIM2** | TTGACCTAAGTGACAACACTGGG | ATGAACTCCAGATGTCAGCTGTAG |
| **ASC** | GCACTTTATAGACCAGCACCG | CTGAAGAGCTTCCGCATCTTG |
| **GAPDH** | CCTCAAGATCATCAgCAATG | CACGATACCAAAgTTGTCAT |

**Table S2.** Primers for polymerase chain reaction in SNP genotyping

| SNP_ID | PCR primer F | PCR primer R | UEP_SEQ |
| --- | --- | --- | --- |
| rs34298354 | ACGTTGGATGGAAACAGCAGATGGAGAGTG | ACGTTGGATGGGCTGCAGCAAACTGGAAAG | CCTTGCCCAGACATC |
| rs3806268 | ACGTTGGATGGATAGTCAAACCTGTCTTGG | ACGTTGGATGGGGATTGGGAAAACAATCCT | GTAGAGTGTCCCCGA |
| rs3804100 | ACGTTGGATGTGCCTGAAACTTGTCAGTGG | ACGTTGGATGTTCCAGTGTCTTGGGAATGC | AGCACACGAATACACAG |
| rs3804099 | ACGTTGGATGTATGCTGCTTCATATGAAGG | ACGTTGGATGGATCTACAGAGCTATGAGCC | TGAAGGATCAGATGACTTAC |
| rs5743705 | ACGTTGGATGTGACTCCATTGAAAAGAGCC | ACGTTGGATGAAATGGGAGAAGTCCAGTTC | CTTTGTGCTTTCTGAAAACTT |
| rs7525979 | ACGTTGGATGTGATGAGCATTCTGAGCCTG | ACGTTGGATGTCCAACATCATCTTCCTGGC | ctTGAGCCTGTGCACAC |
| rs1792773 | ACGTTGGATGCAGTGGTTTCCCAATATGTG | ACGTTGGATGGTGTAAGCTGAAACTGAAAG | AAGGAAGGTAAAAAGAAAGGTG |
| rs2276405 | ACGTTGGATGAGGCCTGGATAACATCACTG | ACGTTGGATGTGCAGTATGTAGTTTGCCTG | tGTTTAAGTTCTTTCTTTCAGAC |

**Table S3. Demographics of participants enrolled for genotyping.**

|  | Controls  (n=119) | MAC-LD  (n=106) | p value |
| --- | --- | --- | --- |
| Age, year | 57.1±15.3 | 65.7±12.6 | <0.001 |
| Male sex | 58 (49) | 42 (40) | 0.170 |
| Smoking |  |  | 0.142 |
| Current | 9 (8) | 2 (2) |  |
| Quit | 12 (10) | 12 (11) |  |
| Diabetes mellitus | 11 (9) | 7 (7) | 0.466 |
| Autoimmune disease | 2 (2) | 5 (5) | 0.182 |
| Cirrhosis of liver | 0 | 2 (2) | 0.132 |
| End stage renal disease | 0 | 1 (1) | 0.219 |
| Active cancer | 0 | 3 (3) | 0.260 |
